# Study Effects of Gradation and Material Thermal Property of Chip Seal Aggregates in Roller Concrete Pavement Crack Healing by Image Processing and RMS

**DOI:** 10.3390/ma16114118

**Published:** 2023-05-31

**Authors:** Zahra Norozi, Mohammad Mehdi Khabiri

**Affiliations:** Department of Civil Engineering, Yazd University, Yazd 8915818411, Iran

**Keywords:** image processing, response surface methodology, crack healing, chip seal, roller concrete pavement

## Abstract

One of the most roller cement concrete pavement failures of pavement is the formation of first cracks. The roughness of its completed surface after the installation has restricted the usage of this pavement. Therefore, engineers increase the quality of service of this pavement by placing a layer of asphalt coating; The primary goal of this study is to evaluate the impact of particle size and type of chip seal aggregates on filling cracks in rolled concrete pavement. Accordingly, rolled concrete samples with chip seal covering were prepared with various aggregates (limestone, steel slag, and copper slag). Then, the influence of temperature on its self-healing ability was tested by putting the samples in the microwave device for cracking improvements. With the aid of Design Expert Software and image processing, the Response Surface Method reviewed the data analysis. Even though due to the study’s limitations, a constant mixing design was applied, the results of this study indicate that the amount of crack filling and repair in specimens slag is higher than that of aggregate materials. With the increase of steel and copper slag, 50% of repair and crack repair at 30 °C, the temperature is 27.13% and 28.79%, respectively, and at 60 °C, the temperature is 58.7% and 59.4%, respectively.

## 1. Introduction

Road and pavement construction are undoubtedly considered the most extensive and essential development activities of any country [1] as well as the first step towardprosperity and region development [2]. Pavement building is an essential aspect of modern living. Cement is one of the most important, and commonly used materials for construction, road building, and cement-containing products emit a substantial quantity of greenhouse gas [3]. Utilizing natural and raw resources causes environmental devastation and disturbance of natural ecosystems [4].

In most countries of the world, flexible pavements always have the largest share in road construction; however, the application of this pavement is accompanied by high environmental hazards and disadvantages, such as instability against atmospheric factors, low resistance to heavy loads, and the need for constant repair and replacement [5]. Many studies have been conducted in the application of concrete types. In the research results, researchers have investigated the effect of different molarities, the percentage of alkali to the binder, and the ratio of sodium silicate to sodium hydroxide on increasing fresh and mechanical properties of concrete. They have paid for geopolymer concrete. They showed that the structural performance of geopolymer concrete is higher than that of traditional concrete [6]. The structural sector, such as columns, and the road construction sector, such as concrete pavement, rely heavily on concrete materials, their damage management, avoidance of cracking, and failure. Cracking is a common disease of concrete structures, and the use of microbial calcite deposition to the repair cracks is the focus of current research [7]. In research for the effective control of crack repair, a comparison of the velocity distribution of elastic waves obtained from acoustic emission tomography analysis was used before and after repair. The results show the potential of the tomographic technique to be used to estimate the healing effect of the injection [8]. Roller concrete pavement is dry (very low water-to-cement ratio) and low-cement concrete, with zero slump, and low hydration heat. The elastic coefficient of roller concrete pavement is less than that of ordinary concrete, which makes it perform better against cracking [9]. Concrete pavement can be used to pave the floor of covered and open cisterns, wood, timber, and coal depots, etc.; cargo terminals, docks, and ports; roads for low-speed heavy vehicles and chained military vehicles, sloping roads, roads with low traffic, and speed; and urban and intercity streets, car parks, and internal traffic lanes of organizations and airports [10,11].

Pavement damage increases rapidly after the operation due to vehicle loads [12]. Timely use of chip seals on old concrete slabs can improve the service level of the pavement and increase its life. In addition to repairing cracks, it also increases the abrasion resistance to improve the concrete surface and is used to make the road surface impermeable. Its use will strengthen the main layer of pavement. Numerous parameters of an asphalt pavement management database are utilized to characterize various road characteristics for various uses. This indicates that some characteristics of the pavement management database do not influence the choice to repair or upgrade the flexible pavement. In the road pavement management system, the proper repair and maintenance of cracks extend the pavement’s life by a quantitatively significant amount. This procedure prevents water penetration into the bottom layers, preserving the pavement’s carrying capacity and preventing its surface from deteriorating [13]. One of the main failures in rigid pavements is cracks; hence, some studies have been performed on cracking concrete pavement, including RCC pavement and its repair [14,15]. Neville, for example, investigated the phenomenon of intrinsic repair of concrete and its cause in 1981. The researcher found that fine cracks in concrete are completely repaired under wet conditions due to hydration delays, the presence of unhydrated cement, and carbonation [16]. Furthermore, several studies have been conducted on using different percentages of cement and additives, such as smelting iron slag and fly ash [17,18]. Research on the effect of fibers on the performance of RCC pavement revealed that the mortar–fiber interaction strongly affects the post-crack roller-compacted concrete pavement (RCCP) behavior. Therefore, post-crack area hardening in the load-crack mouth opening displacement (CMOD) curve is expected for high RCCP flexural strength at a high volumetric fraction of fibers [19]. In recent decades, there has been a rise in the use of waste materials for asphalt, concrete, and building. The most significant emphasis has been paid to recycled concrete aggregates, recycled asphalt paving aggregates (RAP), crumb rubber, and steel slag aggregates from the electric arc furnace, which are extensively employed. According to previous research on the use of recycled materials in concrete, the tensile and compressive strengths of concrete are reduced due to the inclusion of these elements. However, little research has been conducted on using these materials in RCC for road pavement. There is still a great deal of research to be carried out on the properties of rolled concrete containing recycled materials [20]. To study the effect of roller-compacted concrete (RCC) mixed waste materials with RAP or crumb rubber as a partial replacement of aggregates on mechanical properties, different samples with waste contents from 25 to 100% RAP and 5 to 25% crumb rubber were made in the laboratory. Then, these mixtures were tested to determine the composition efficiency of these wastes in concrete pavements. The results showed that the compressive and flexural strength of RCC specimens is improved by 5% rubber content. In addition, a combination of up to 10% rubber and 50% RAP could be cost-effective and useful to increase the pavement life by increasing the toughness and energy absorption of mixtures [21]. In a study, the effect of shrinkage cracks on static load-bearing capacity and fatigue performance of RCC-reinforced pavements with recycled steel fibers were evaluated. It was found that these fine cracks penetrate up to a quarter of the slab thickness and reduce load-bearing capacity and pavement fatigue capacity by up to 50%. Even if the contraction initially causes minor cracks, they are significantly intensified due to the traffic load [22]. The mechanical performance of RCC containing crumb rubber and nano-silica was studied by evaluating sixteen laboratory samples, including four different percentages of crumb rubber (0, 10, 20, and 30%) and nano-silica (0, 1, 2, and 3%). Results showed that increasing the crumb rubber increases the consistency, compressive strength, slip resistance, and tensile strength, and adding nano-silica improves the performance of RCC as well [23].

The crack behavior of RCC mixtures containing modified asphalt pavement, and crumb rubber were analyzed by testing a set of 288 semicircular cracked bending (SCB) specimens with asymmetric three-point bending. Results revealed that the characteristics of the concrete mixture have a significant effect on the onset of RCC cracking [11]. Research on the fracture properties of RCC reinforced with hybrid fibers manifested that the addition of fibers to the RCC mixture fails to affect the occurrence of possible torsion cracks during crack propagation. Results of the three-point bending test on shear beams showed a positive synergistic effect of short steel and macro polypropylene fibers on fracture toughness [24].

Fresh and mechanical properties, as well as the durability of RCC containing regenerated asphalt pavement aggregates, were evaluated in the laboratory. The RCC mixture containing 50% coarse RAP and 50% fine aggregate was found to have high flexural strength. In addition, RCC mixtures containing recycled asphalt had sufficient abrasion resistance and were suitable for construction in areas with high concentrations of chloride and sulfate ions. Based on the results, higher ratios of fine-grained RAP were suitable for RCC application in sulfate environments [25]. The effect of polypropylene fibers on fractur, and mechanical performance of RCC pavements was examined by performing a three-point bending test on grooved beams with different thicknesses. The fibers failed to improve the compressive and flexural strength, however, significantly improved the behavior of after cracking [26].

The utilization of Response Surface Methodology (RSM) enables the establishment of links between various experimental factors and their corresponding responses through the integration and examination of multiple experimental designs. The tensile strength ratio of dry and saturated hot mix asphalt was evaluated by researchers using RSM to examine the impact of aggregate gradation and lime content. Using RSM, researchers identified the modifications in the rheological characteristics of asphalt binder at high temperatures due to varying amounts of Sasobit. Certain researchers employed RSM to investigate the influence of aggregate gradation, hydrated lime, and Sasobit content on the indirect tensile strength of warm mix asphalt. Additionally, other researchers identified the optimal conditions for incorporating plastic waste into asphalt mixing, utilizing RSM to enhance performance in terms of resistance to mixture failure under repetitive loading [27]. In pavement detection, the Image Processing Method (IPM) has found extensive applications. To identify different types of pavement distress, various pavement inspection devices have been developed, such as digital cameras for detecting pavement cracks and laser scanning methods for identifying rutting or cracks, as demonstrated by researchers. Furthermore, Thermal Imaging Technology is currently utilized for controlling temperature segregation during asphalt paving. Additionally, image technology has been implemented for the extraction of pavement texture information for detecting potholes and joint faulting. Furthermore, owing to the progress and evolution of image detection technology, image systems have been integrated into vehicles to identify pavement failure and distress [28]. Previous studies have demonstrated the successful utilization of RSM and IPM, indicating their capacity to delineate the complex behavior of pavement and its rehabilitation.

It was discovered that the surface condition of the road pavement is one of the safety factors. Previous studies have discussed the impact of surface deterioration on the safety of the pavement. According to the findings of the studies on the influence of repairing cracks on increasing friction and the safety of vehicle movement, it is crucial to repair cracks. Therefore, this experimental investigation aims to use appropriate materials and stone grading to the self-repair of pavement cracks. The roughness of the finished surface after execution has faced limitations and problems in applying this pavement. Thus, engineers improve the service quality of this pavement by applying a layer of asphalt coating. One of the most critical defects of asphalt-coated roller pavement is cracking; hence, the primary purpose of this study is to investigate the effects of gradation and aggregate material in filling RCC pavement cracks. In summary, the findings of this study may be used for the selection of slag materials and their proper grading, resulting in a reduction in the cost of making slag chip seal. In addition, these findings provide the foundation for large-scale laboratory manufacturing and real-world applications of this self-healing response.

## 2. Materials and Methods

**Figure 1:**
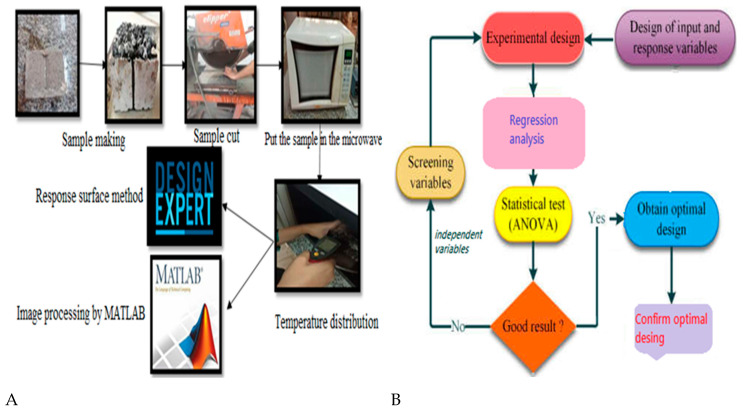
Flowcharts of the (**A**) research process, (**B**) Response Surface Method.

In this study, to evaluate the type of aggregation and aggregate material type in filling RCC cracks, first, samples of RCC containing a chip seal coating with aggregation and different aggregates, such as lime, steel slag, and copper slag, were made. Then, the samples were placed in the microwave to evaluate the crack improvement. The research variables are temperature, type, and grading of chip seal aggregates, and the dimensions of the crack opening. Two types of grading, C and D, were selected from the standard of the chip seal mixing design; D grading is coarser than C, but both are relatively fine-grained. Image processing was used to check the degree of filling or crack healing. Results were analyzed through the response level method, Design Expert Software, as well as image processing. A visual flowchart of the overall research process and RSM is provided in Figure 1.

### 2.1. Materials Used

**Figure 2:**
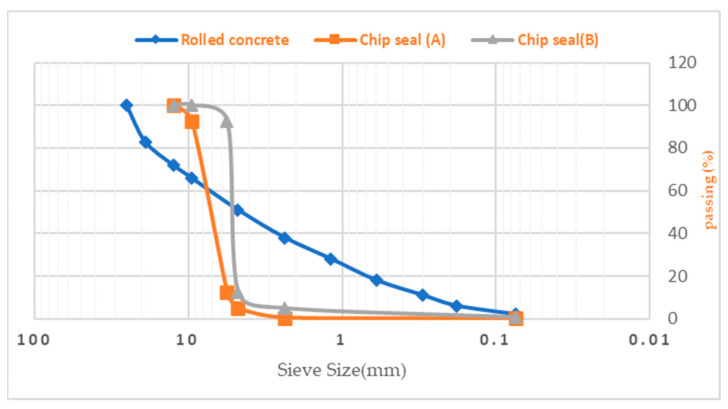
Gradation used.

The gradation presented in Figure 2 was used to make roller and chip seal concrete. According to the ASTM D6913/D6913M-17 standard procedure, sieving is used to assess the grain size distribution of soils with a particle size larger than 0.075 mm [29]. Further, the characteristics of bitumen according to the ASTM D2397 standard and aggregates used in the chip seal are presented in Table 1 and Table 2, respectively [30,31].

### 2.2. Sample Preparring

**Figure 3:**
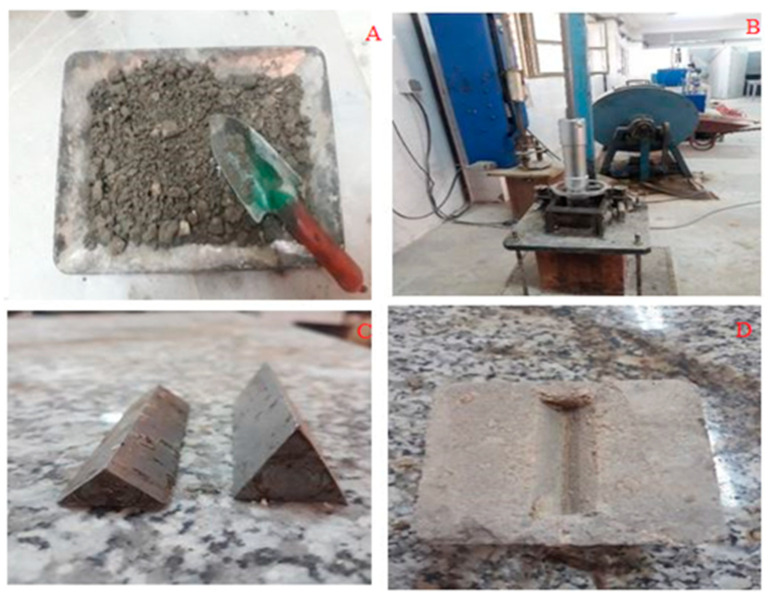
(**A**). Concrete sample mixture, (**B**). Density of concrete mixture, (**C**). Steel blade to create cracks in the sample. (**D**). RCC sample.

In this study, to make samples, first, RCC samples were prepared according to the ASTM D1557 standard [32] and then steel blades with different dimensions were used to create cracks (Figure 3). To spread the chip seal on rolled concrete samples, first, its surface was thoroughly cleaned, covered with the calculated amount of emulsion bitumen, and then aggregates were spread on it in the calculated amount. The thickness of the layer is 12.5 mm (half an inch), because emulsion bitumen does not create thickness, and the layer thickness is equal to the dimensions of the largest aggregate.

**Figure 4:**
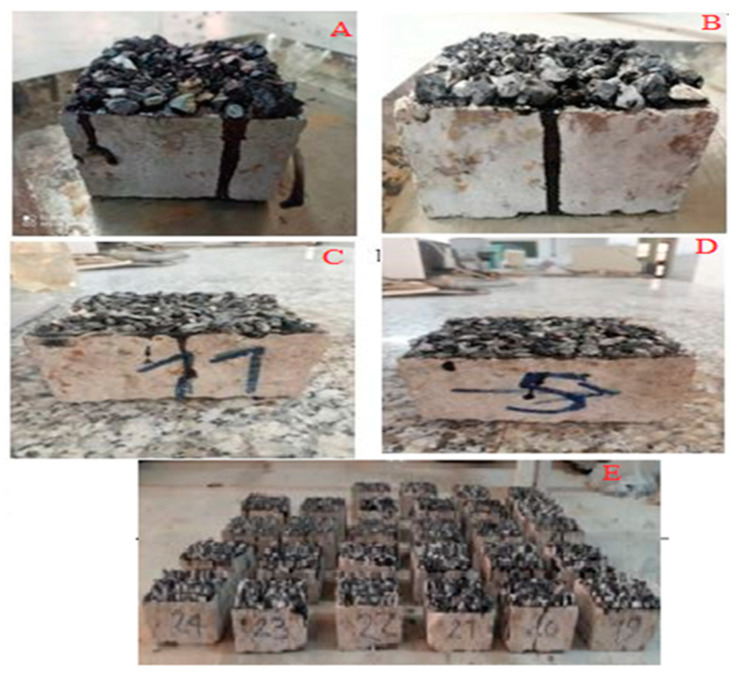
(**A**). Chip seal containing copper slag with type C gradation on the concrete sample with larger crack depth, (**B**). Chip seal containing copper slag with type C gradation on the concrete sample with smaller crack depth, (**C**). Chip seal containing steel slag with type D gradation on the concrete sample with smaller crack depth, (**D**). Chip seal containing steel slag with type D gradation on the concrete sample with larger crack depth, (**E**). All samples made for testing.

After making the RCC sample, a chip seal layer was applied to the RCC sample. As mentioned, three types of aggregates, including lime aggregate, steel slag, and copper slag, were used in the construction of the chip seal asphalt mixture. If coarse electric arc furnace slag that substitutes aggregate has reduced porosity and water absorption, the mechanical characteristics will be enhanced. If its porosity and water absorption are relatively high, it will reduce the mechanical qualities of concrete. With the incessant exploitation of primary high-quality mineral resources, these resources could be regularly exhausted, so it is of great meaning to recover metal basic oxygen furnace (BOF) slag as subordinate mineral resources. According to Table 2, it is also evident that copper and iron metals exist in the slag as oxides and were used in this research. Hence, 30, 40, and 50% of the material left on the one-half inch (6.3) mm sieve in the type A grain chip seal and 30, 40, and 50% of the materials remaining on the grade 4 sieve in the type B chip seal gradation were replaced with steel slag and copper slag (Figure 4). Samples were named according to Table 3.

### 2.3. Cutting Samples and Putting in the Microwave

**Figure 5:**
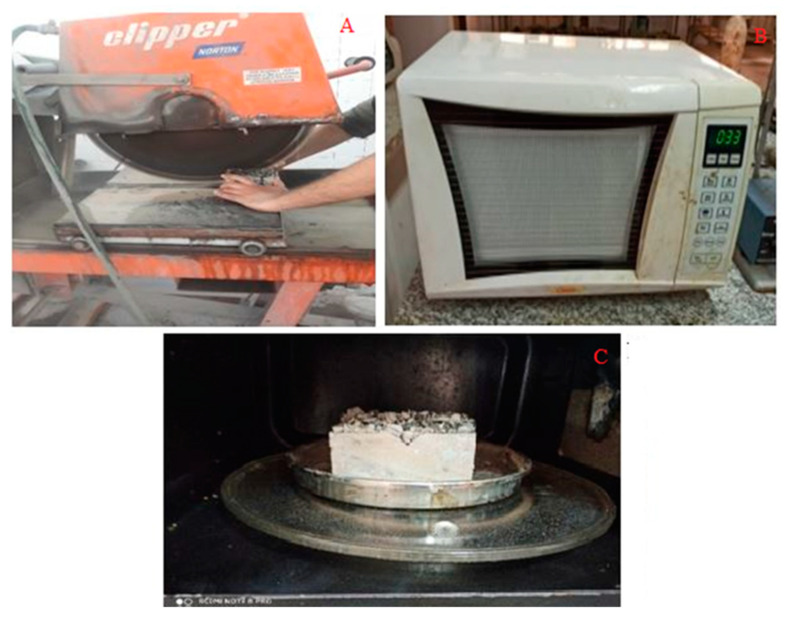
(**A**). Cutting the sample using a stone saw and cut sample, (**B**). Microwave device to put the sample inside it, (**C**). The sample placed in the microwave device.

To observe both deep and surface cracks created on the concrete sample, it was first cut from the middle by a stone saw, according to Figure 5A, and then placed in a microwave at two different temperatures of 30 and 60 °C (Figure 5B,C). Since asphalt pavement temperatures are often selected between 30 and 60 °C in experiments and studies, and temperatures beyond this range seldom occur on the pavement surface, these temperature ranges were employed in this investigation for crack-healing performance [32]. Since laboratory equipment has temperature control constraints, one of the limitations of using a microwave device is the limitation of precise temperature control with an accuracy of ±1 degree Celsius and other small dimensions of the interior space of the microwave. In this study, smaller rolled concrete samples were selected and the temperature inside the microwave was measured and regularly controlled with a laser thermometer.

In addition, based on the mixing design used in the manufacturing of RCC (fixed mixing design), the findings of this research may be relevant within the scope of this mixing design. In addition, not all employed slag materials include the same proportion of minerals and there may be subtle variations in the slag’s chemical components.

### 2.4. Temperature Distribution

**Figure 6:**
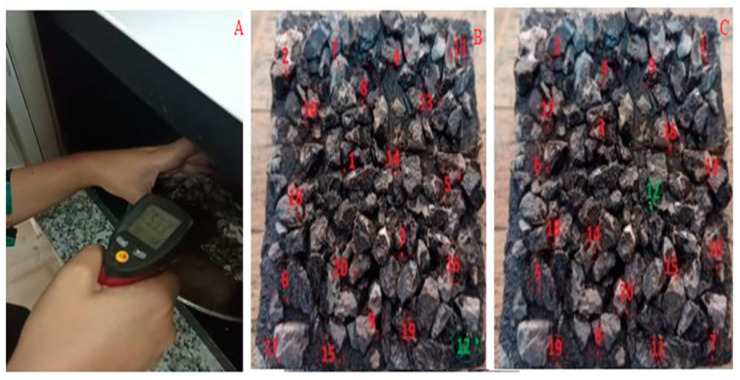
(**A**). Temperature measuring method, (**B**). Temperature distribution points recorded with a laser thermometer in the sample at a temperature of 30 and (**C**). 60 degrees.

A part of the experiments in this research includes examining the capability of asphalt samples by the method of electromagnetic heating waves and their induction repair at different temperature levels. In order to achieve the highest thermal recovery rate, different samples were placed under-regulated heating for a certain period. In this way, each concrete sample with the seal chip coating was exposed to microwave radiation for 120 s. Then, the temperature of the sample was measured by a laser thermometer from inside the microwave. The temperature of five points on the sample’s surface was measured and averaged randomly. Finally, the surface temperature at two temperatures of 30 and 60 degrees with a tolerance of 1 degree Celsius was the criterion for further studies. The error of the laser thermometer used was 0.05 degrees Celsius. Using a stone saw, 28 samples were divided into two parts, which yielded a total of 56 samples. Since bitumen acts like a Newtonian fluid between 30 to 60 °C, half of the samples were placed in the microwave at 30 °C and the other half at 60 °C. Then, based on the amount of repair in the type of cracks, the surface temperatures of each sample at 20 points were measured using a laser thermometer (Figure 6).

### 2.5. Response Surface Method

RSM is a set of mathematical methods determining the relationship between one or more response variables and several independent (studied) variables. This method was introduced by Box and Wilson in 1951 and is still used as an experimental design tool [33,34].

To present a mathematical model between independent and dependent parameters using the RSM, after selecting the equation scheme, the model is determined and its coefficients are predicted. The model used in the RSM is generally the equation of a complete quadratic model or its reduced form. The quadratic model can be expressed as Equation (1) in which $\beta_{0}،\;\beta_{j}،\;\beta_{jj}\;و\;\beta_{ij}$ are considered as constant, linear, quadratic, and regression interaction coefficients, respectively, and $X_{i},\;\mathrm{and}\;X_{j}$ as independent variables. Matrix symbolization is given in Equations (2) and (3) [33,34]:

(1) $$Y=C_{k0}+\sum_{i=1}^{4}C_{ki}x_{i}+\sum_{i=1}^{4}C_{kii}x_{i}^{2}+\sum_{i<j=2}^{4}C_{kij}x_{i}x_{j}$$

(2) y=Xβ+ε

(3) $$\left[\begin{array}{c} y_{1}\\ y_{2}\\ .\\ .\\ .\\ y_{n} \end{array}\right]=\left[\begin{array}{c} 1\;x_{11}\;x_{12}\;\ldots \;x_{1k}\\ 1\;x_{21}\;x_{22}\;\ldots \;x_{2k}\\ \ldots \ldots \ldots \ldots \ldots \ldots \ldots \ldots \ldots \ldots \\ \ldots \ldots \ldots \ldots \ldots \ldots \ldots \ldots \ldots \ldots \\ \ldots \ldots \ldots \ldots \ldots \ldots \ldots \ldots \ldots \ldots \\ 1\;x_{n1}\;x_{n2}\;\ldots \;x_{nk} \end{array}\right]\left[\begin{array}{c} \beta_{0}\\ \beta_{1}\\ .\\ .\\ .\\ \beta_{k} \end{array}\right]+\left[\begin{array}{c} \varepsilon_{1}\\ \varepsilon_{2}\\ .\\ .\\ .\\ \varepsilon_{n} \end{array}\right]$$

The above Equations (1)–(3) are obtained using the solved least squares method and equation coefficients. After obtaining equation coefficients, the answer is predicted by solving the above equation. The conformity of the model with the experimental data should then be checked. There are some methods for doing this, such as residual analysis, root mean square of predicted errors, and mismatch test. The overall predictability of the model is expressed by the determination coefficient (*R*^2^) and its statistical significance by the Fisher test (F-Value). The significance of each regression coefficient is obtained based on the *t*-test as well. However, *R*^2^ alone fails to explain the accuracy of the model as this indicator represents changes in the mean response; hence, another coefficient called the adjusted coefficient of explanation ($R_{\mathrm{adj}}^{2}$) is used to calculate, which, unlike *R*^2^, the average sum of squares is used instead of the sum of squares. Equations (4) and (5) show how these two coefficients are calculated [35].

(4) $$R^{2}=1-\frac{SS_{residual}}{SS_{total}{}_{}}$$

(5) $$R_{adj}^{2}=1-\frac{SS_{residual}/DF_{residual}}{SS_{total}/(DF_{\mathrm{model}}+DF_{residual})}$$

${SS}_{residual}$ = The sum squares residual
*DF =* Degrees of freedom
${SS}_{total}$ = The sum of the total squares $({SS}_{residual}+{SS}_{model}$)

As mentioned, in this study, the RSM and Design Expert Software were used to investigate the effect of different parameters on the cracks’ repair in RCC pavement with the chip seal coating. For this purpose, the parameters of grain type, material of chip seal aggregates, temperature, and crack depth were considered independent variables, and sample surface temperature, which indicates self-healing ability, was considered a dependent variable.

### 2.6. Image Processing by MATLAB

**Figure 7:**
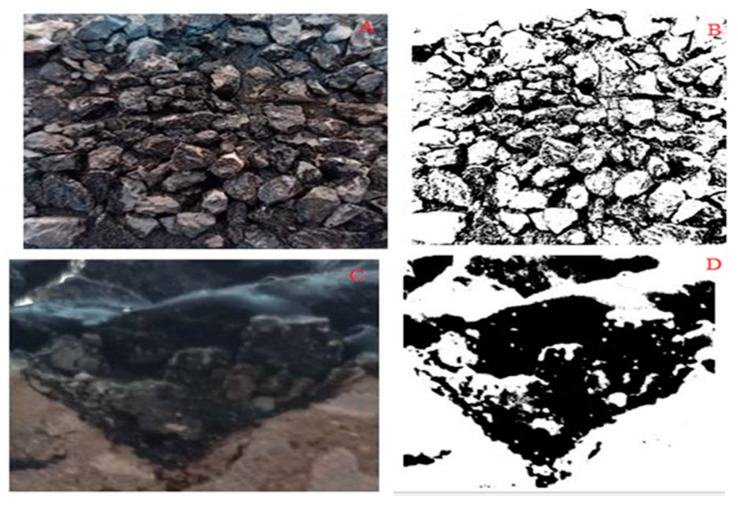
Image taken from the sample surface ((**A**). Before, and (**C**). After cutting), and converted to black and white photo (**B**,**D**).

For evaluating the surface porosity and amount of filling and crack healing, images of the surface of the samples at the laboratory temperature (26 °C) and laboratory samples cut at the ambient temperature 30 and 60 °C were prepared and then examined by MATLAB Software (MATHWORKS MATLAB R2019a v9, MathWorks, MA, USA). Using this software, the prepared color images turned into black and white images so that the aggregates inside the asphalt turned white and the rest black. The software then calculates the ratio of the number of white pixels to the total number of pixels, which is called Brightness. An example of these images is given in Figure 7.

## 3. Results and Discussion

### 3.1. Temperature Distribution Results

**Figure 8:**
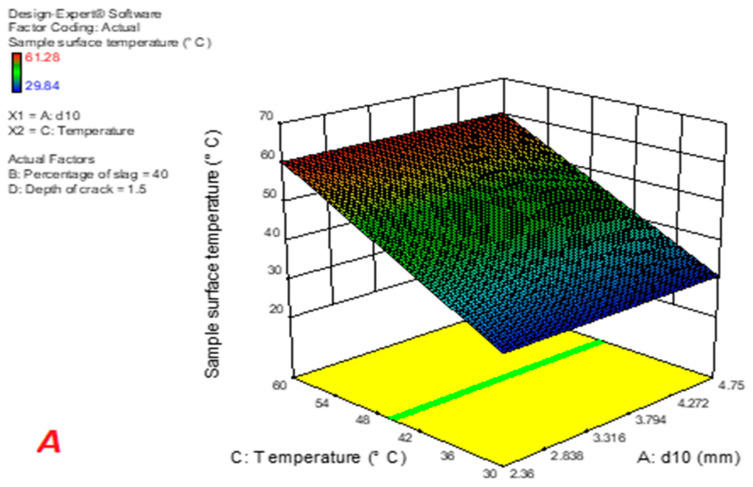

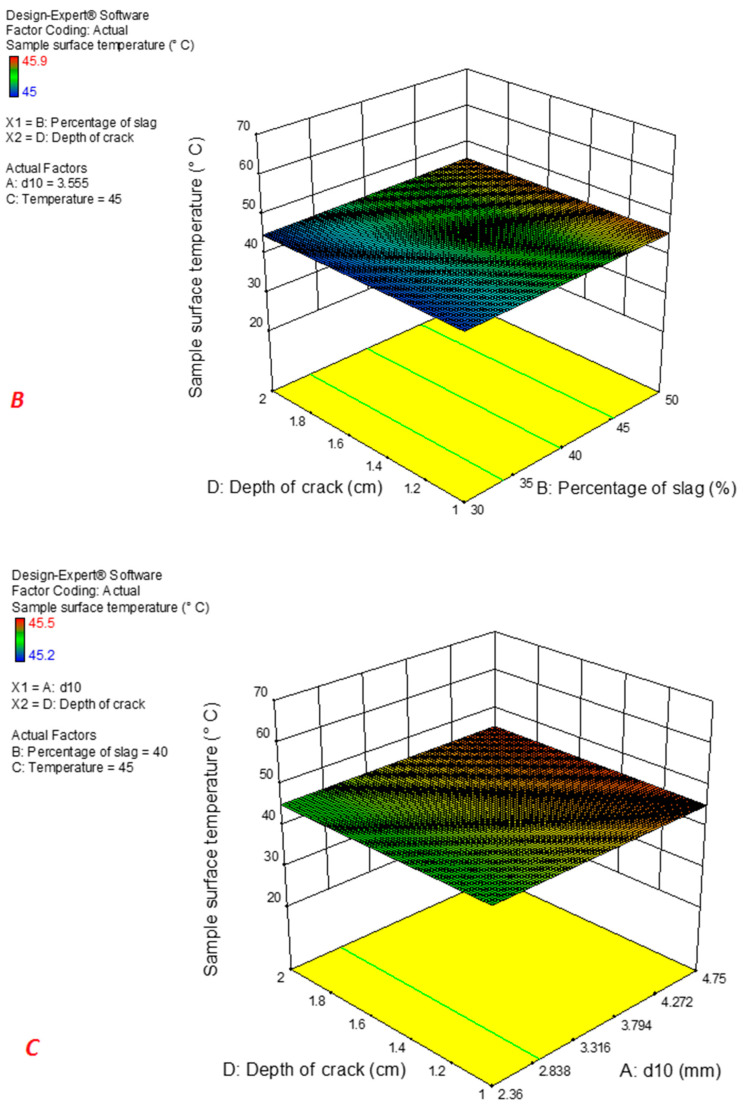
Average temperature in samples made of copper slag changing: (**A**): the d_10_ and temperature; (**B**): the slag amount and the crack depth; (**C**): the d_10_ and crack depth.

**Figure 9:**
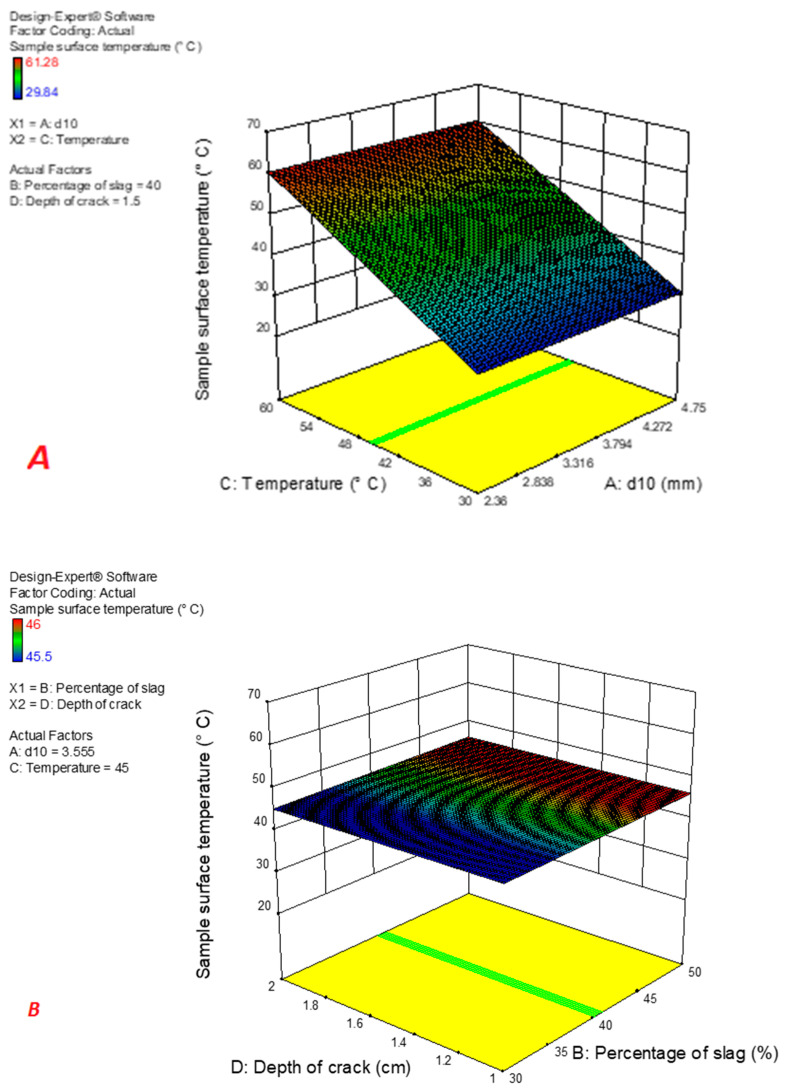

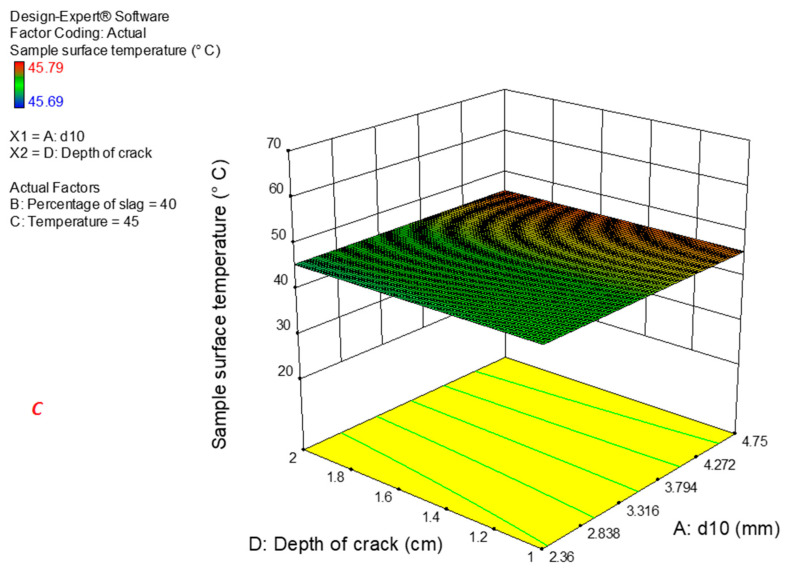
**Figure 9.** Average temperature in samples made of copper slag changing: (**A**): the d_10_ and temperature; (**B**): the slag amount and the crack depth; (**C**): the d_10_ and crack depth.

The results of measuring the temperature distribution in the samples with different gradations and aggregates are described in Table 4 and Table 5. The average temperature of the samples below 30 and 60 °C is also presented in Figure 8 and Figure 9.

The influence of temperature is one of the most crucial self-healing characteristics. In a study on crack healing, the optimal temperature for bitumen repair during the resting time and without loading was found to be 50 to 60 °C [36].

As Figure 8 and Figure 9 show, the samples containing slag had a higher temperature in the microwave with temperatures of 30 and 60 °C compared to the control samples, the reason for which can be the use of slag and heat absorption by it, leading to an increase in the sample temperature. Due to this, the cracks had better self-healing than the control samples. In addition, at a certain percentage of slag, samples containing copper slag had a higher temperature than samples containing similar steel slag, which could be due to better thermal conductivity and a higher percentage of iron oxide in copper slag than steel slag (Table 4 and Table 5).

### 3.2. Determining the Relationships between the Percentage of Slag and Temperature of Surface

Based on Table 6, the relationship between the surface temperature of the sample containing steel slag and parameters of gradation, slag percentage, temperature, and crack depth is shown in Equation (6). Table 7 shows the results of the ANOVA analysis (SPSS22, IBM, NY, USA) of this relationship. According to the F-value, which indicates the effect of each parameter on the desired response, the percentage of slag and type of gradation have the most significant effect on the samples’ surface temperature, respectively. Moreover, since the *p*-value of the gradation parameters, slag percentage, and temperature is less than 0.05, these parameters have a significant effect on the surface temperature of the sample.

Sample surface temperature = 1.642 − 0.03056 × d_10_ − 1.3608 × 10^−3^ × Percentage of slag + 0.93989 × Temperature + 0.01523 × Depth of crack + 4.7071 × 10^−4^ × d_10_ × Percentage of slag + 1.02859 × 10^−3^ × d_10_ × Temperature + 4.7071 × 10^−3^ × d_10_ × Depth of crack + 7.8869 × 10^−4^×Percentage of slag × Temperature − 2.32143 × 10^−4^ × Percentage of slag × Depth of crack − 4.5833 × 10^−4^ × Temperature × Depth of crack (6)

To examine the effect of parameters on the dependent variable as accurately as possible, the software coded Equation is used (Equation (7)).

Sample surface temperature = +45.43 + 0.05 × A + 0.35 × B + 14.62 × C + 1.027 × 10^−3^ × D + 5.625 × 10^−3^ × AB + 0.018 × AC + 2.813 × 10^−3^ × AD + 0.12 × BC − 1.16 × 10^−3^ × BD − 3.438 × 10^−3^ × CD (7)

In this Equation:

A = d_10_
B = Percentage of slag
C = Temperature
D = Depth of crack

According to Equation (7), with increasing slag percentage, the average surface temperature of the sample increases, resulting from the presence of iron oxide in steel slag, which increases the absorption and thermal conductivity of the sample. By Table 8, the relationship between the surface temperature of the sample containing copper slag and gradation parameters, slag percentage, temperature, and crack depth is presented in Equation (8). The results of the ANOVA test regarding this relationship are displayed in Table 9. As F values manifest, the percentage of slag and gradation type have the greatest effect on the surface temperature of the samples, respectively. In addition, since the *p*-value of the slag percentage and temperature parameters is less than 0.05, these parameters have a significant effect on the sample surface temperature.

Sample surface temperature = +1.48818 + 3.362 × 10^−1^ d_10_ + 6.08936 × 10^−5^ × Percentage of slag + 0.94225 × Temperature + 0.050976 × Depth of crack − 3.5116 × 10^−4^ × d_10_ × Percentage of slag + 5.7531 × 10^−4^ × d_10_ × Temperature + 5.75314 × 10^−3^ × d_10_ × Depth of crack + 1.0125 × 10^−3^ × Percentage of slag × Temperature − 8.92857 × 10^−3^× Percentage of slag × Depth of crack − 1.54167 × 10^−3^ × Temperature × Depth of crack (8)

To examine the effect of parameters on the dependent variable more precisely, the software-coded Equation is used (Equation (9)).

Sample surface temperature = 45.76 + 0.025 × A + 0.44 × B +14.74 × C − 7.589 × 10^−4^ × D − 4.196 × 10^−3^ × AB + 0.01 × AC + 3.437 × 10^−3^ × AD + 0.15 × BC − 4.464 × 10^−4^ × BD − 0.012 × CD (9)

In this Equation:

A = d_10_
B = Percentage of slag
C = Temperature
D = Depth of crack

According to Equation (9), with increasing slag percentage, the average surface temperature of the sample increases, which can be due to the presence of iron oxide and copper oxide in copper slag, increasing thus the thermal absorption and thermal conductivity of the sample. Applying Table 10, the relationship between the surface temperature of the sample containing steel slag and parameters of gradation, slag percentage, temperature, and crack depth is presented in Equation (9). The results of the ANOVA test regarding this relationship are displayed in Table 11.

### 3.3. Image Processing

**Figure 10:**
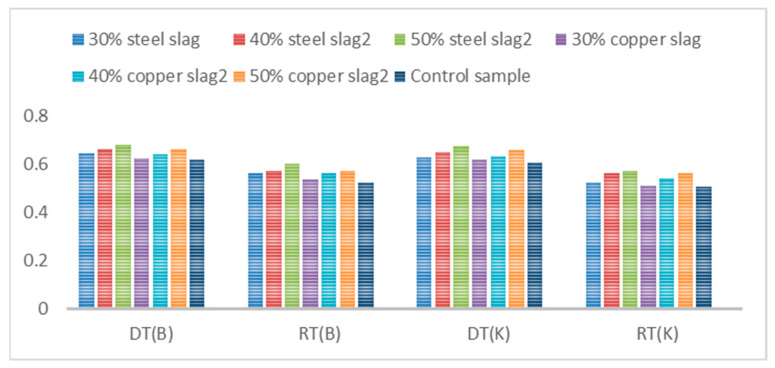
Surface porosity of samples before cutting.

**Figure 11:**
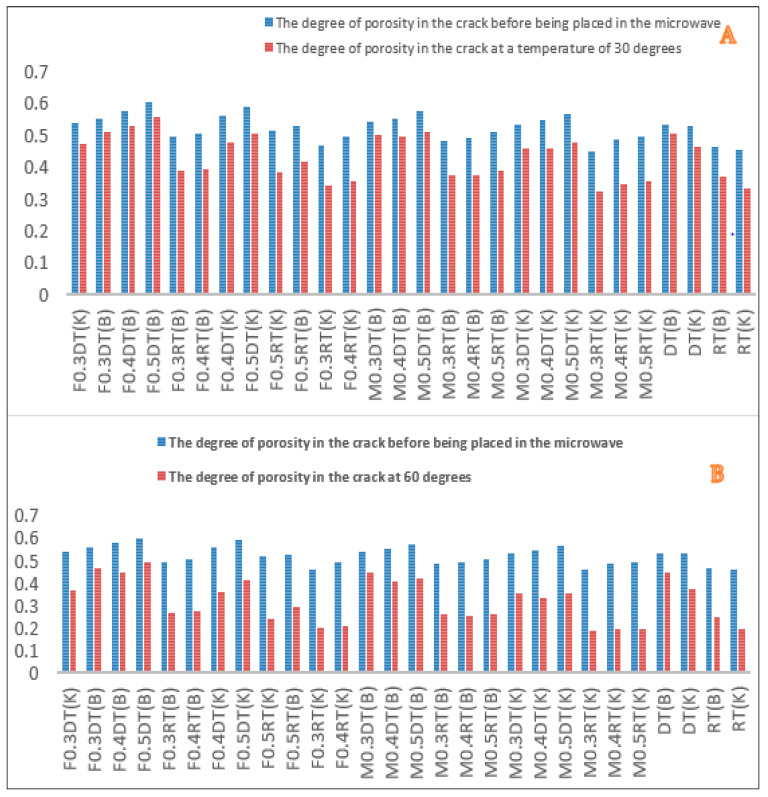
Porosity calculated by MATLAB software for cut samples before putting in the microwave and rate of porosity, (**A**). After being exposed to 30 °C, (**B**). After being exposed to 60 °C.

As mentioned, in this study, using image processing, the amount of porosity, improvement, and filling in the crack was investigated before and after cutting, the results of which are shown in Figure 10 and Figure 11.

As shown in Figure 10, the use of metal slags has increased the surface porosity of the samples compared to the control samples, which have increased with the increase of slag percentage. This can be due to the porous texture of slags and adsorption of bitumen by them. Furthermore, by peer-to-peer comparison of samples, it can be seen that in a specific type of gradation and cracking, samples containing steel slag have a higher porosity than those with copper slag. This is due to the more porous texture of steel slag compared to copper slag since bitumen absorbs the pores of steel slag and fills less space in the mixture. In addition, in a certain percentage of metal slag, coarse-grained samples have higher porosity because the amount of porosity in the coarse grains is more than the fine-grained ones. On the other hand, as can be seen in Figure 10 in a specific type of gradation, copper percentage, and steel slag, the amount of surface porosity in samples with larger cracks is higher than in samples with more minor cracks, indicating a low impact of cracks in increasing porosity. Furthermore, the use of conductive fibers and filaments promotes crack healing (Crack Repairing), which in turn increases fatigue resistance (Cracking Factor) [37,38].

**Figure 12:**
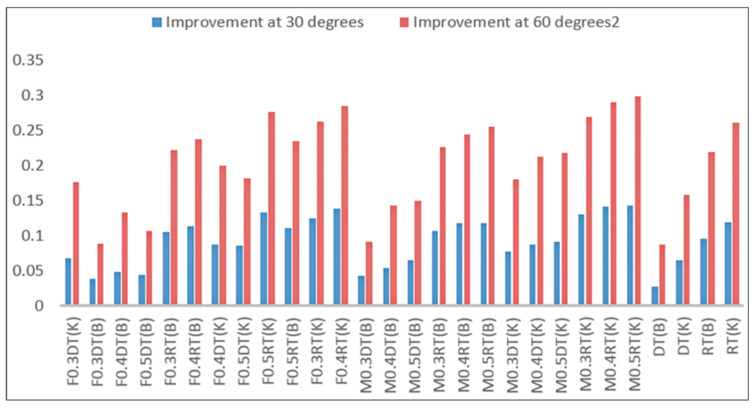
Improvement of sample porosity.

According to the diagrams in Figure 11, the amount of porosity in the cracks of the cut samples before being placed in the microwave is higher than that of the sample at 30 and 60 °C, indicating the improvement of cracks at these temperatures. Figure 12 represents the rate of porosity and crack recovery at 30 and 60 °C. Further, it manifests that the rate of crack recovery in samples exposed to 60 °C is much higher than in samples exposed to 30 °C; because at a temperature of 60 degrees, bitumen is smoother and penetrates the pores and cracks, making the cracks heal faster.

Additionally, the use of metal slags has increased the rate of recovery and crack filling at temperatures of 30 and 60 °C, and this rate of improvement increases with increasing the percentage of slags (Figure 12). Moreover, in a certain percentage of metal slag, samples with finer grains have a higher rate of improvement than samples with larger grains and similar cracks. This due to the lower porosity in samples with finer gradation because of better locking and tightening of aggregates. In a specific gradation and crack, samples containing copper slag have a higher recovery rate than those with steel slag due to better thermal conductivity and greater heat absorption. On the other hand, in a type of gradation and a certain percentage of slag, samples with smaller cracks have a higher rate of improvement than samples with larger cracks. When high thermal conductivity metal particles (copper or steel) in the slag are subjected to microwaves, they begin to absorb heat. This energy transfer is passed to the neighboring bitumen molecules and atoms. These bituminous molecules are distributed more rapidly in concrete discontinuities and fractures to fill them. Grading (grain size) also allows it to fill up tiny fissures at the commencement of cracking. Due to their greater specific surface area, smaller pebbles may absorb and transmit heat energy to the bitumen surrounding them faster.

RCC is a low-cost, long-lasting pavement that has attracted considerable interest in the pavement business. In addition, it is possible to extend its life by repairing tiny and minor cracks using chip seal, a low-cost coating, which may be used to remove small and minor cracks. Therefore, the Turks’ self-repair via this way seems to be an economic issue. Utilizing trash and slag is an additional economic argument for this process. By selecting the proper slag type and grading for various temperature circumstances, it is feasible to achieve a favorable economic performance with the aid of the findings of this study.

## 4. Conclusions

One of the most critical failures of roller pavement is cracking; thus, the primary purpose of this study is to investigate the effects of gradation and aggregate material in filling RCC pavement cracks. The results of this research are as follows:

- In samples containing coarse-grained and fine-grained chip seals with an increase of steel slag by 50%, the average surface temperature of the sample at 30 °C increased by 3.95 and 3.83%, and at 60 °C by 4.15 and 4%, respectively, compared to the corresponding control samples.
- In samples containing coarse-grained and fine-grained chip seals with an increase of copper slag by 50%, the average surface temperature of the sample at 30 °C increased by 4.74 and 4.84%, and at 60 °C by 5 and 5.5%, respectively, compared to the corresponding control samples.
- With an increase of steel slag by 50% in concrete samples with larger and smaller cracks and coarse-grained chips, the average rate of crack recovery and the filling at 30 °C increased by 7.61 and 15%, at 60 °C by 18.92 and 32%, and in the corresponding control samples at 30 °C by 5 and 12.24%, and at 60 °C by 15.44 and 29.15%, respectively.
- In samples containing fine-grained chip seals and larger and smaller cracks with a 50% increase in the steel slag, the average crack recovery and filling at 30 °C were 21.61 and 27.13%, respectively, at 60 °C, 45 and 57.8% in the corresponding control samples, at 30 °C, 20.3 and 26%, and at 60 °C, 42 and 56%.
- The recovery rate and crack filling were higher in samples containing copper slag and at a temperature of 60 °C. Furthermore, in the samples with fine-grained chip seals and smaller cracks, the rate of recovery and filling was higher than in those with coarse-grained chip seals and larger cracks.

Using copper and steel slag to produce chip seals, and in the crack healing process of concrete surface cracks, is a novel concept that not only reduces the consumption of raw materials and ecological harm, but also improves the condition of the concrete pavement structure and avoids the formation of cracks. This concept is proposed for a future field study over a longer time in regions with varying temperatures and environmental conditions. In addition, owing to the potential of greater heat and energy absorption by metal oxides (slag), the possibility of melting surface ice and lowering the likelihood of freezing in the concrete pavement should be covered with a chip seal, which requires more research.

## Figures and Tables

**Table 1:** Specifications of aggregates and bitumen used according to the ASTM D2397 method.

Bitumen Type		Bitumen Test	
CRS-1			
43		Viscosity by Saybolt Furol Viscometer at 50 °C (s)	
12		Bitumen Percentage	
Maximum 1		Stability Against Sedimentation	
Maximum 1%		Remaining on the sieve	
Maximum 12		Volumetric Percentage of Emulsion Oil in Distillation Test	
115		Remaining bitumen test from distillation	Penetration degree (one tenth of a millimeter)
40			Ductility property
99.7			Solubility in trichloroethylene (%)
Specifications of limestone Aggragtes			
Test results	Allowed according to MS-2	Description	
18	40	Maximum wear by the Los Angeles method (percentage) ASTM C131/AASHTO T96	
7.14	30	Maximum flakiness index by BS 812 method (percent) AASHTO T96	
98		Fracture percentage on one side ASTM D5821	
97	80	Minimum percentage of breakage on both sides on sieve number 4 ASTM D5821	
1.4	2.5	Maximum water absorption percentage (coarse aggregates) ASTM D5821	
1.6	2.8	Maximum water absorption percentage (fine-grained materials) AASHTO T85	
2.667	-	Specific weight of coarse aggregates ASTM C127	
2.711	-	Specific weight of fine aggregates ASTM C128	
2.645	-	Specific weight of filler ASTM C188	
2.14	-	Percentage of filler water absorption ASTM C128	

**Table 2:** Characteristics of steel and copper slag aggregates.

Characteristic	Amount
Steel slag characteristics	
Specific weight (gr/cm^3^)	3.31–3.56
Los Angeles abrasion (%)	29.5
Decrease in sodium sulfate product (%)	0.2–0.5
Sand equivalent (%)	65.8
Two-sided fractures (%)	90
Fe_2_O_3_	24%
SiO_2_	16%
Al_2_O_3_	8.3%
MgO	2.21%
Cao	35%
Loss on Ignition	14.49%
Copper slag specifications	
SO_3_	0.11
Cr_2_O_3_	0.17
BaO	0.26
P_2_O_5_	0.07
TiO_2_	0.27
MoO_3_	0.06
SiO_2_	33.29
CaO	3
ZnO	0.5
Al_2_O_3_	5
K_2_O	0.79
CuO	2.2
MgO	2.1
Cl	0.02
Fe_2_O_3_	50.5
PbO	0.16
Loss on Ignition	1.5

**Table 3:** Coding different samples in the research.

Sample Code/Name	Type of Crack	Aggregate Type
F0.5DTB	Large opening crack	Coarse grain containing 50% steel slag
F0.3DTK	Small opening crack	Coarse grain containing 30% steel slag
F0.4RTB	Large opening crack	Fine grain containing 40% steel slag
M0.5DTB	Large opening crack	Coarse-grained containing 50% copper slag
M0.3DTK	Small opening crack	Coarse grain containing 30% copper slag
M0.4RTB	Large opening crack	Fine grain containing 40% copper slag
RTK	Small opening crack	Fine grain without slag

**Table 4:** Temperature distribution in different parts of the samples at a temperature of 30 °C.

Point Numbers	Temperature at Different Points of the Samples						
	F0.3DT_K_	F0.4RT_B_	M0.3DT_K_	M0.4RTB	RTK	F0.5DTB	M0.5DTB
1	29.5	29.72	30	30.1	29.3	30	32
2	31	31.1	29.9	30.6	30.3	29.5	30
3	30.3	29.9	30.4	29.6	29.3	30.2	30.8
4	30.5	31.6	31	30	30.6	31.3	31.6
5	30.2	30.4	31.2	31	30	29.9	30.4
6	30.7	30.1	30.5	30.4	29.7	30.1	30.2
7	31.2	30.9	30	31.3	30.1	31.8	33.5
8	31	30.5	30.9	29.5	30	32.1	31.7
9	29.9	30	30.7	30.3	29.7	30.8	30.7
10	30	31.2	30	31.7	30.1	31.8	32.5
11	31.4	30.5	31.5	32	30.2	31.1	30.4
12	31.7	31.9	31.7	32.4	30.9	32.4	33.3
13	30.7	30.1	30.3	30.1	30.5	32.2	30.3
14	31	30.6	30.7	30.8	30	31.5	30.5
15	30.8	30.2	31.8	30.9	28.8	30.4	30
16	30.5	31.3	30.6	31.1	29.2	31.9	32.1
17	30	31.7	30.1	32.1	29.9	30.5	31.3
18	30.8	30.4	30.8	31.2	30.4	31.5	30.9
19	30.1	30.7	31.3	31.4	29.5	31.3	32.7
20	30	30.7	31.2	32.2	28.7	32.5	33

**Table 5:** Temperature distribution in different parts of the samples at a temperature of 60 °C.

Point Numbers	Temperature at Different Points of the Samples						
	F0.3DT_K_	F0.4RT_B_	M0.3DT_K_	M0.4RT_B_	RT_K_	F0.5DT_B_	M0.5T_B_
1	56.3	61	60	61.9	55.5	60	61
2	57	60	61.1	60	58.7	61.5	60
3	58.5	56.9	56.9	58	60	61.2	60.8
4	60	58.5	58.6	62	56	58.7	62
5	56	60.3	61	56.7	60	59.5	63.7
6	60	61.9	59.9	60.9	55.9	62	58.8
7	60.1	56.5	60	57.2	60.1	58.3	60.5
8	59.5	60.6	57.2	61.5	56.9	60.9	61.3
9	61	60.4	61.7	60.7	58	62.9	63
10	59	57.9	60.2	59.7	59	58.5	59
11	60.9	59.9	56.8	62.4	56.5	60.3	60
12	61.3	62	61.9	62.1	60.3	62.5	63.5
13	60.4	61.5	60.8	58.8	57.4	59.2	60.4
14	57.7	57.3	60.4	60.5	58.4	60.7	62.9
15	60.2	60	60.3	61.2	59.5	59.9	61.7
16	61.2	60.8	57.8	60.3	57.3	63	59.3
17	61.4	59.1	61.6	60.2	58.1	61.7	61.1
18	60.6	60.2	61.5	57.2	60.6	58.8	63.3
19	61.2	62.1	60.5	61.6	56.3	61.7	60.3
20	61.3	60.5	61.5	60.4	57.5	61.8	62.5

**Table 6:** Model summary statistic to Equation (6).

	R-Squared	R-Squared	*p*-Value	Source
	0.9996	0.9997	<0.0001	Linear
Suggested	1.0000	1.0000	<0.0001	2FI
Aliased	1.0000	1.0000	0.6293	Quadratic

**Table 7:** ANOVA analysis related to Equation (6).

*p*-Value	F Value	Mean Square	df	Sum of Squares	Source
<0.0001	90268.19	674.17	10	6741.69	Model
0.0092	8.23	0.061	1	0.061	A-d10
<0.0001	1885.16	14.08	1	14.08	B-Percentage of slag
<0.0001	7.119 × 10^−5^	5317.03	1	5317.03	C-Temperature
0.9533	3.513 × 10^−3^	2.624 × 10^−3^	1	2.624 × 10^−5^	D-Depth of crack
0.4985	0.47	3.544 × 10^−3^	1	3.544 × 10^−3^	AB
0.2409	1.46	0.011	1	0.011	AC
0.8557	0.034	2.531 × 10^−4^	1	2.531 × 10^−4^	AD
<0.0001	209.88	1.57	1	1.57	BC
0.8883	0.020	1.509 × 10^−4^	1	1.509 × 10^−4^	BD
0.8241	0.051	3.781 × 10^−4^	1	3.781 × 10^−4^	CD
		7.469 × 10^−3^	21	0.16	Residual
			31	6741.85	Cor Total

**Table 8:** Model summary statistics to Equation (8).

	R-Squared	R-Squared	*p*-Value	Source
	0.9994	0.9995	<0.0001	Linear
Suggested	0.9999	0.9999	<0.0001	2FI
Aliased	0.9999	0.9999	0.0276	Quadratic

**Table 9:** ANOVA analysis related to Equation (8).

*p*-Value	F Value	Mean Square	df	Sum of Squares	Source
<0.0001	41584.08	683.19	10	6831.94	Model
0.3441	0.94	0.015	1	0.015	A-d10
<0.0001	1334.30	21.92	1	21.92	B-Percentage of slag
<0.0001	3.290 × 10^5^	5405.49	1	5405.49	C-Temperature
0.9767	8.725 × 10^−4^	1.434 × 10^−5^	1	1.434 × 10^−5^	D-Depth of crack
0.7324	0.12	1.972 × 10^−3^	1	1.972 × 10^−3^	AB
0.6537	0.21	3.403 × 10^−3^	1	3.403 × 10^−3^	AC
0.8809	0.023	3.781 × 10^−3^	1	3.781 × 10^−4^	AD
<0.0001	157.24	2.58	1	2.58	BC
0.9709	1.359 × 10^−3^	2.232 × 10^−3^	1	2.232 × 10^−5^	BD
0.6152	0.26	4.278 × 10^−3^	1	4.278 × 10^−3^	CD
		0.016	21	0.35	Residual
			31	6832.29	Cor Total

**Table 10:** Model summary statistics to Equation (9).

	R-Squared	R-Squared	*p*-Value	Source
	0.9994	0.9995	<0.0001	Linear
Suggested	0.9999	1.0000	<0.0001	2FI
Aliased	0.9999	1.0000	0.0012	Quadratic

**Table 11:** ANOVA analysis related to Equation (9).

*p*-Value	F Value	Mean Square	df	Sum of Squares	Source
<0.0001	41584.08	683.19	10	6831.94	Model
0.3441	0.94	0.015	1	0.015	A-d10
<0.0001	1334.30	21.92	1	21.92	B-Percentage of slag
<0.0001	3.29 × 10^5^	5405.49	1	5405.49	C-Temperature
0.9767	8.725 × 10^−4^	1.434 × 10^−5^	1	1.434 × 10^−5^	D-Depth of crack
0.7324	0.12	1.972 × 10^−3^	1	1.972 × 10^−3^	AB
0.6537	0.21	3.403 × 10^−3^	1	3.403 × 10^−3^	AC
0.8809	0.023	3.781 × 10^−4^	1	3.781 × 10^−4^	AD
<0.0001	157.24	2.58	1	2.58	BC
0.9709	1.359 × 10^−3^	2.232 × 10^−5^	1	2.232 × 10^−5^	BD
0.6152	0.26	4.278 × 10^−3^	1	4.278 × 10^−3^	CD
		0.016	21	0.35	Residual
			31	6832.29	Cor Total

## Data Availability

Data supporting this study is included within the article.
